# Menopausal Symptoms and Dementia Risk

**DOI:** 10.1002/alz70857_100868

**Published:** 2025-12-24

**Authors:** Sophie Alderman, George Stothart, Martha Hickey, Nicholas Turner, Elizabeth Coulthard

**Affiliations:** ^1^ University of Bath, Bath, United Kingdom; ^2^ University of Melbourne, Melbourne, Australia; ^3^ University of Bristol, Bristol, United Kingdom; ^4^ Bristol Medical School, University of Bristol, Bristol, United Kingdom

## Abstract

**Background:**

Identifying sex‐specific risk factors for Alzheimer's disease (AD) is essential as two‐thirds of cases are women, with female sex being one of the biggest risk factors for AD after increasing age (Subramaniapillai et al., 2021). A longer life expectancy was originally thought to be a key contributor to this overrepresentation, but after controlling for survival rates in men and women, the increased risk has been shown to persist (Rocca et al., 2014). Recent work has demonstrated a link between frequent nocturnal vasomotor symptoms (hot flushes/night sweats: VMS), the cardinal menopausal symptom, and AD blood‐based biomarkers (Thurston et al., 2024). This study aims to build on this research by investigating the mechanisms that underpin this association through the use of plasma biomarkers, objective measures of VMS and electroencephalography (EEG) to detect sleep macroarchitecture and assess cognition.

**Hypotheses:**

Severe nocturnal VMS will be positively associated with increased AD pathology and those experiencing severe symptoms will show changes in sleep macro architecture. We also predict severity of symptoms will be negatively associated with cognitive function.

**Methods:**

We will recruit 150 perimenopausal and postmenopausal women experiencing VMS symptoms. The Stages of Reproductive Aging Workshop (STRAW + 10) will be used to classify participants according to their menopausal status (Harlow et al., 2012) and COMMA (Core Outcomes in Menopause) measurements (Lensen et al., 2023) will be used to assess symptom severity and interference. All participants will wear an EEG headset for 7 nights to measure sleep and VMS symptoms will be measured using an ambulatory monitor. Participants will also complete a cognitive assessment battery and provide a blood sample for biomarker analysis.

**Results:**

This is the research study design. The study is due to begin in October 2025.

**Conclusion:**

The project aims to establish whether nocturnal VMS could be a female‐specific risk factor for AD. Identifying severe and frequent VMS as a midlife risk factor for women could result in the early introduction of interventions which could help to preserve brain health and reduce the incidence and rate of cognitive decline.

